# Numerical study of a highly efficient light trapping nanostructure of perovskite solar cell on a textured silicon substrate

**DOI:** 10.1038/s41598-020-75630-4

**Published:** 2020-10-29

**Authors:** Alireza Tooghi, Davood Fathi, Mehdi Eskandari

**Affiliations:** 1 Department of Electrical and Computer Engineering, Tarbiat Modares University (TMU), Tehran, Iran; 2 Nanomaterial Research Group, Academic Center for Education, Culture and Research (ACECR) on TMU, Tehran, Iran

**Keywords:** Solar cells, Solar energy and photovoltaic technology

## Abstract

In this paper, a nanostructured perovskite solar cell (PSC) on a textured silicon substrate is examined, and its performance is analyzed. First, its configuration and the simulated unit cell are discussed, and its fabrication method is explained. In this proposed structure, poly-dimethylsiloxane (PDMS) is used instead of glass. It is shown that the use of PDMS dramatically reduces the reflection from the cell surface. Furthermore, the light absorption is found to be greatly increased due to the light trapping and plasmonic enhancement of the electric field in the active layer. Then, three different structures, are compared with the main proposed structure in terms of absorption, considering the imperfect fabrication conditions and the characteristics of the built PSC. The findings show that in the worst fabrication conditions considered structure (FCCS), short-circuit current density (J_sc_) is 22.28 mA/cm^2^, which is 27% higher than that of the planar structure with a value of 17.51 mA/cm^2^. As a result, the efficiencies of these FCCSs are significant as well. In the main proposed structure, the power conversion efficiency (PCE) is observed to be improved by 32%, from 13.86% for the planar structure to 18.29%.

## Introduction

In recent years, organometal halide perovskite solar cells (PSCs) have received much attention and have made exceptional progress in terms of energy conversion efficiency^1–3^. Within a few years, the PCE of these cells has increased from 3% in 2009^4^, to more than 22% in 2019^5^. Features such as appropriate absorption in the visible spectrum^6^, long diffusion length of carriers^7^, high carrier mobility^8^, easy manufacturing^9^, and low production costs^10,11^ have made perovskite a promising nominee for use as light absorbent material in next-generation solar cells.

PSCs are conventionally designed in two general architectures, planar and mesoporous. In recent years, the use of nanostructures with different dimensions and shapes in the construction of various types of solar cells resulted in high efficiencies with less volume of absorbing material and generally more complex cell structures^12–17^. The use of nanostructures causes the cell to be lighter, more flexible and less bulky, in addition to enabling improvements in efficiency. Nanostructures enhance cell performance through the mechanism of light trapping in the absorbing medium^18^, improving carrier extraction^19^, or concentrating the electromagnetic field in the active layer using various configurations, especially plasmonic structures^20^.

In light trapping structures, due to a shift in the direction of light path within the active layer, its interaction time with this layer increases, which increases the optical absorption^21–24^. In these structures, by scattering the light to the off-normal directions, in addition to prolonging its pathlength in the active layer, the probability of reflection of each interface increases due to its angular movement. Therefore, light passes through the absorbing layer via multiple paths, which increases likelihood of absorption^25^. Depending on the wavelength of the incoming wave, and the dimensions and formation of the nanostructure, different light trapping mechanisms may occur within the solar cell^26^.

In plasmonic structures, plasmonic resonance occurs at the interface of metallic and non-metallic mediums by the collective excitation of the metal surface electrons gemerated by the electromagnetic waves^27,28^. Depending on the dimensions of the metal nanostructures (MNS) used in the main structure, the surface plasmon resonance (SPR) would happen in one of two mechanisms - near-field or far-field. If the size of the MNS were small (<50 nm), the SPR has mainly near-field effects and causes the electromagnetic field to concentrate in the vicinity of the MNS. But if the size of these nanostructures were larger (>50 nm), the SPR is mostly of the far-field scattering type and the plasmonic structure acts as a back or forward scatterer^29^. In 2015, Carretero-Palacios et al. analyzed the effect of the size and concentration of Au nanoparticles (NPs) embedded within the perovskite film on its absorption and have demonstrated that in such a configuration, the absorption is maximized when near-field and far-field effects of SPR are balanced^30^. Cui and co-workers reported a 13.7% improvement in efficiency using Au NPs inside the CH_3_NH_3_PbI_2.85_Br_0.15_ perovskite solar cell, which is related to the local enhancement of the electric field due to the near-field effects of SPR^31^. In 2018, Shi et al. introduced a high performance perovskite light-emitting diode with a core-shell nanostructured configuration and Au NPs inside the structure of the photovoltaic device, and achieved an emission enhancement ratio of 1.55 due to local surface plasmon coupling^32^.

There are various methods for shaping micro and nanostructures used in micro sensors, micro actuators, and nano devices. Among them, silicon micro-machining technologies have made great strides in recent decades^33^. This is due to the valuable properties of silicon, such as precisely shaping capability, compatibility with technologies used in the fabrication technologies of the integrated circuits, broad availability, and suitable electrical, mechanical, and thermal properties. Therefore, these advanced technologies could also be used in the fabrication of nanostructured solar cells^34^.

In this work, we use modeling studies to analyze the performance of nanostructured PSC on a textured silicon substrate. First, conventional planar PSC is simulated to verify the applicability of the modeling method used in this research. The obtained electrical outputs are set as references for the proposed structure. The optical losses of planar structure, which cause energy loss in this cell and reduction of its efficiency are investigated. The light trapping structure of the PSC based on a silicon substrate is presented, in which a PDMS layer is used as a cell protector instead of glass to reduce light reflection. The fabrication method of such a cell, based on an etched crystalline silicon substrate, is explained in this paper, and the simulation model used in this research is also discussed. The optical properties of this structure are compared with those of the planar structure considering the electric field profiles and the geometric path of light inside the cell. The effect of decreasing the density of hole transport material on the PSC performance according to the occurred plasmonic resonance is investigated. Then, three possible shapes of the structure concerning the deposition limitations and fabrication conditions are considered and compared with the ideal structure to estimate its efficiency after construction. Finally, the electrical properties of the main structure and three structures in which fabrication conditions were considered are investigated and compared.

## Results

**Figure 1:**
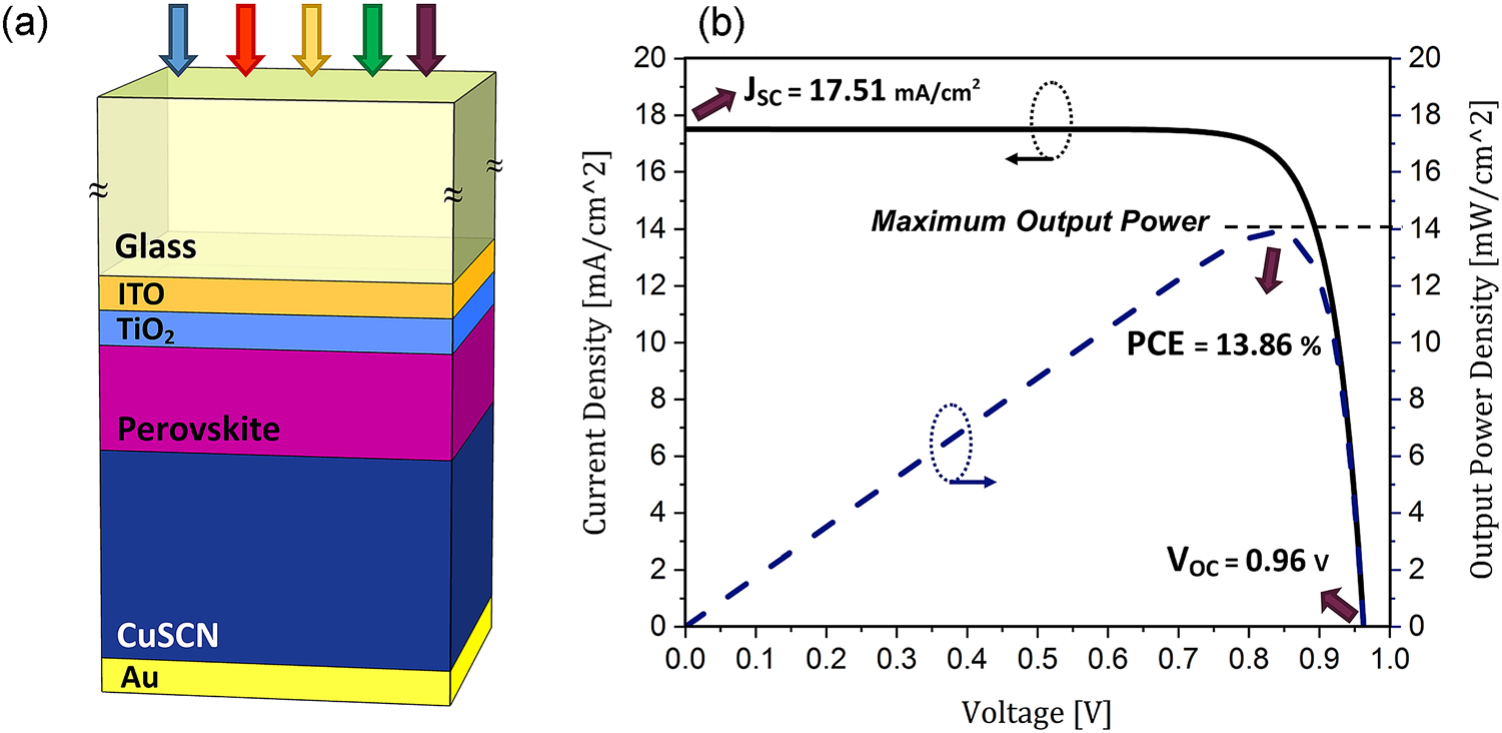
(**a**) A schematic of a planar structure of a PSC. (**b**) The current-voltage, and output power-voltage characteristics of a planar PSC.

Figure 1a shows the typical planar architecture of a perovskite solar cell. This structure comprises five layers located on a flat glass substrate. These layers are ITO (indium doped thin oxide), TiO_2_ (titanium dioxide), Perovskite (methylammonium lead iodide), CuSCN (copper thiocyanate) and Au with the thicknesses of 50, 50, 200, 600, and 100 nm, respectively. The properties of perovskite, which is used as the absorbing material, has been discussed before. Here, TiO_2_ is used as the electron extraction and hole blocking layer. It is widely used in PSCs due to its high chemical stability, appropriate optical and electronical properties, and simple deposition methods^35,36^. In this structure, CuSCN is used as the hole transport layer (HTL). It is a cheap and efficient inorganic p-type material. PSCs based on this architecture have high efficiency and thermal stability in addition to having low fabrication costs. CuSCN possesses desirable properties such as high mobility, transparency to visible light, and suitable energy band levels^37^.

The output parameters of this structure obtained using the computational model are described in methods section and are used as the reference structure for comparison with subsequent structure. Figure 1b shows the current and output power density characteristics of this planar structure in terms of voltage. According to this figure, J_sc_, open-circuit voltage (V_oc_), PCE, and filling factor (FF) for this structure are 17.51 mA/cm^2^, 0.96 V, 13.86%, and 82.42%, respectively. The results obtained in this section are in good agreement with the results of previous numerical and experimental studies for a planar PSC with similar dimensions^13,37,38^.

**Figure 2:**
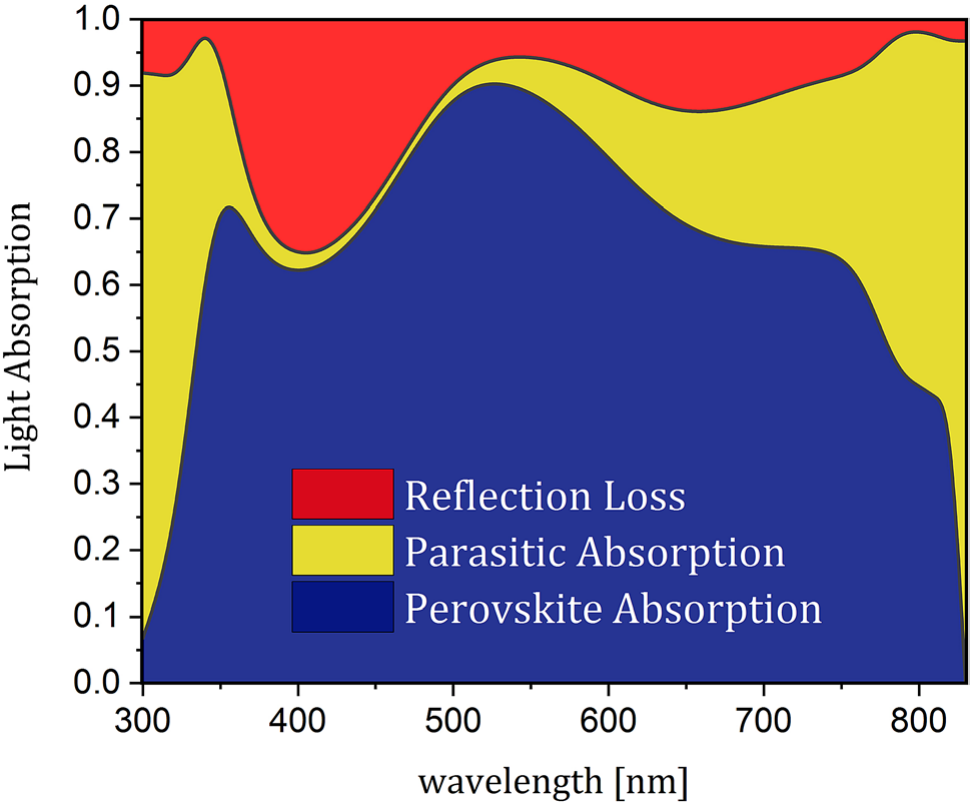
The spectrum of the perovskite absorption, parasitic absorption of the other layers, and total reflection of a planar PSC.

Figure 2 presents the light absorption scheme of the perovskite, parasitic absorption of other layers, and the total reflection in terms of the impact light wavelength for the planar structure of a PSC. This spectrum shows that not all of the energy of the incoming light is absorbed in the active layer, and a considerable amount of the energy is dissipated. According to calculations, 20% of light is lost due to parasitic absorption of the other layers and 13% to reflection. Since the carrier recombination during transportation in the cell is insignificant due to the low thickness of the perovskite and high diffusion length of carriers, it does not limit the output current. Therefore, the low J_sc_ in the planar PSC is due to the limited light absorption in the perovskite layer. Thus, to increase the current density and output power corresponding to it, the amount of energy loss in the cell must be reduced. For this reason, the light trapping structure is used to facilitate the entry of light into the cell by reducing the reflection, and to confine light to the absorbing layer by reducing the parasitic losses of the inactive layers.

### Presentation of the proposed nanostructured perovskite solar cell

**Figure 3:**
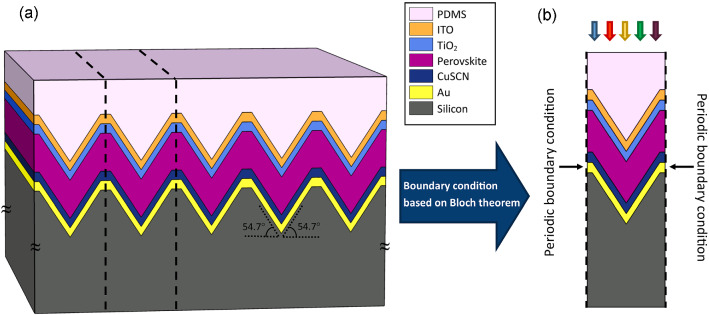
(**a**) A schematic of the proposed nanostructure of a PSC on an etched silicon substrate, and (**b**) the unit cell of the computational domain used in optical simulation with applied periodic boundary condition.

Figure 3a shows our proposed structure for a perovskite solar cell. In this structure, due to the specific fabrication process, a thin layer of PDMS with a thickness of 100 nm is used instead of the thick glass layer. PDMS in addition to being a transparent material with nearly no absorption in the visible and near-infrared spectral range, enhances the physical resistance of the cell and reduces reflection from the surface due to its suitable refractive index. Furthermore, a cell with this structure is more flexible than the conventional planar structure because of the absence of a thick layer of glass. The layers of this cell must be deposited on a textured silicon substrate to shape the presented light trapping structure.This structured solar cell, unlike the planar structures, should be built from the bottom layer, as described in the following section.

#### Studying the structural formation and simulated unit cell of the proposed PSC

Bulk micro-machining technique using wet anisotropic etching can etch silicon to the desired structure. Using this method, one can make three-dimensional shapes depending on the crystalline orientation of silicon and the type of etchant with the advantages of low cost, high etching speed, and a high degree of selectivity. Since the <100> plane of the crystalline silicon etches more rapidly and at a higher rate than the <111>, by etching the silicon crystal (100), angular shapes can be produced. This angle, which is 54.7 degrees, is the angle between the <100> and the <111> planes and is caused by the fact that the etching is much slower in the <111> plane. Thus, with appropriate orientation of crystalline silicon, choice of suitable etchant and etching time, and use of appropriate temperature, the proposed structure can be obtained with a smooth surface.

Photolithography and wet etching techniques have commonly been used to fabricate V-shaped and pyramidal structures on silicon substrates^39^. In this method, initially, a Si_3_N_4_ film is grown on a silicon (100) wafer, usually by an evaporation method, and then a photoresist (PR) film is deposited by spin coating. After the forming the desired pattern on the PR using photolithography, first the Si_3_N_4_ etching barrier selectively is etched using the buffered hydrofluoric (HF) acid etchant, and then the PR photo-mask is completely removed. In the following, by immersing the wafer in the KOH solution at temperatures between 80 and 85$^{\circ }$C, the desired V-shaped structure with flat surfaces and tips is formed. In the end, to completely remove the Si_3_N_4_, the sample is immersed in diluted hydrogen fluoride^34,39–41^.

Since the proposed PSC must be built from the bottom layer to shape the desired light trapping structure, preserving the quality of the deposited films on the silicon substrate is critical. In order to achieve desired high efficiency, we can utilize fabrication methods used to assemble inverted perovskite solar cells (IPSC). This is because investigations have shown that in IPSCs, in which, the coating of the films is done from the bottom layer, high efficiencies can be obtained using appropriate deposition methods^42–44^. In this regard, with one step fast deposition-crystallization, one can fabricate a high-quality perovskite film on CuSCN layer with low surface roughness and small series resistance. In this process, the thickness of the CuSCN film can be adjusted by varying its electrodeposition time^45^. Additionally, by utilizing the Brookite-phase TiO_2_ top buffer on perovskite film, in addition to better extraction and transport of the carrier, high efficiency and long lifetime IPSC can be obtained^46^.

There are various methods such as evaporation, vacuum deposition, atomic layer deposition, sputtering, etc. to fabricate a conformal perovskite film and other components of the PSC on a textured substrate^47–52^. In this regard, one can use a two-step hybrid technique combining co-evaporation and spin-coating to deposit perovskite on an underlying textured silicon wafer^50^. Studies have also shown that the sputter-based process can be used to fabricate homogeneous perovskite films on a surface with high roughness^53^. Furthermore, if a dry two-step deposition proceeding is used, the quality of perovskite film can be adjusted by controlling the precursor composition, temperature, and duration of the conversion process^34^.

Given that our proposed structure is different from the planar structure, to equalize the light absorption conditions for investigating these two structures, the volume of the absorbing layer in these structures is set to be equal. By this, the carrier generation conditions in these structures would be identical since the generation of electron and hole pairs occurs only in this layer. The layers of the ITO, TiO_2_, and Au also have similar thicknesses in the two structures. However, the CuSCN layer must be thin. This is because, due to the deposition conditions, less the amount of material used in the deposition, the better it follows the form of the underlying layer. However, due to the difficulty of the material deposition with small thicknesses, the CuSCN thickness is taken to be 50 nm, which can be deposited by the method presented in previous research^54^. In order to control the thickness and morphology of the deposited layers, especially perovskite, in addition to the preceding mentioned methods, various techniques, including thermal vacuum deposition, solvent solvent extraction (SSE), ultra-sonic spray-coating, etc. have been proposed by researchers^55–57^. In these suggested deposition methods, the intention is to obtain stable, uniform and smooth films with a desired thickness. Figure 3a shows the periodical structure of the proposed nanostructured PSC. To reduce the amount of computation, as seen in Fig. 3b, a unit cell of periodical structure is considered. The boundary condition, which applied to its sides, is periodic and is based on Bloch’s theorem. The simulation of finite element method (FEM) electromagnetic optical is performed for this computational domain of the unit cell.

### Studying the optical features of the proposed nanostructured PSC

**Figure 4:**
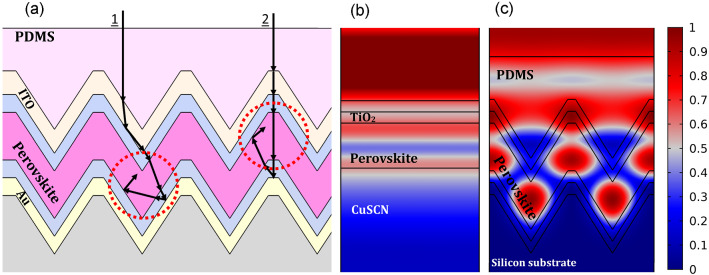
(**a**) A schematic of light path inside a PSC with the proposed structure. (**b**) The normalized electric field profile of the planar and (**c**) presented structures at the wavelength of 800 nm.

Figure 4a shows the geometrical path of moving light inside the PSC with the given structure. Unlike in the planar architecture, in which, the direction of light in the PSC is normal to the cell surface, in this structure, due to the deformation of the configuration, the path of light changes on passing through each layer. In the conventional planar structure, assuming vertical illumination of light to the surface of the cell, the direction of light at the boundary of two layers does not change. But due to differences in the refractive indices of the two media, part of the impact light reflects, and the rest of it goes to the next layer without changing direction. But in this configuration, according to the Snell’s law, due to the angular incident of light at the interface of two media with different refractive indices, the direction of light changes at each layer, and its path is no longer perpendicular to the cell surface.

As presented in Fig. 4a, the light path inside the presented light trapping structure has two states depending on which part of the cell it enters. If the incoming light is closer to the middle of the studied unit cell, according to the state 1, the light path does not change after entering the PSC from the external medium (air). However, at the PDMS/ITO interface, because of the larger refractive index of ITO, it converges slightly to the middle of the cell. At the TiO_2_ interface, due to the high angle of impact, light is reflected, and consequently, approaches the center. Therefore, it reaches the interface of the perovskite at an almost perpendicular angle and thus penetrates it. The direction of light does not change much on passing through HTL. As it collides with the Au back-contact, due to the SPR effect, which will be discussed further in the electric field review section, it scatters back into the absorbing layer. Again, due to the approaching impact angle to the total reflection angle at the interface of perovskite and CuSCN, it returns to the active layer. Thus, light is trapped inside the absorbing layer and moves further inside this layer. This increases the probability of the perovskite absorption and carrier generation inside it. State 2 shows the geometrical light path when it is away from the center of the unit cell. In this case, light moves in a similar path to that of a planar structure, and its direction does not shift much until it reaches the Au back-contact. When it hits the metal surface, due to the angular impact and the scattering effect of surface plasmon resonance, it scatters to the sides of the cell. Therefore, light returns to the absorbing layer at a nonvertical angle. Like state 1, in the perovskite/HTL interface, due to the high impact angle, light is reflected and is trapped in the active layer. Therefore, in both cases, the light that enters the cell is trapped inside the absorbing layer, until it is absorbed.

Figure 4b,c show the profiles of the normalized electric field at wavelength of 800 nm for a planar PSC and the presented structure. At this wavelength, the energy of photons is lower than that of the shorter wavelengths of the perovskite absorption spectrum, and therefore light moves longer before being absorbed. Thus, the electric field profile at 800 nm, in contrast to the lower wavelengths in which light is not trapped completely inside the active layer, is compatible with the light trapping mechanism of the nanostructure. Accordingly, at this wavelength, the comparison of the electric field profiles is accomplished. As expected, in the planar structure, the electric field intensity along the width of the PSC is constant. This indicates that the direction of light in this structure is unaltered and it always moves perpendicular to the cell surface. Also, within the perovskite and near its boundary with the HTL, the electric field intensity is higher than in the middle of the layer. This proves that part of the light reflects into the perovskite from this boundary and enhances the electric field in this area, and part of it enters the lower layer of CuSCN and causes parasitic losses.

One can see that in the proposed structure, the intensity of the electric field in ITO is higher than that of the planar structure, indicating more light entering the cell due to the use of PDMS and the specific configuration of the PSC. This is because PDMS has a closer refractive index to the outer environment of the PSC (air), and the adaptation of the refractive indices of these two adjacent media, reduces the reflection from the cell surface. Unlike the planar structure, in this structure, due to the non-vertical movements of the light, the profile of the electric field is changed completely, and it is no longer constant across the width of the cell. According to this profile, the electric field intensity inside the absorbing medium and in certain areas of it is increased, which shows that light is trapped inside this layer. Part of this trapped light within the perovskite is located in the lower portion of the triangular section, which is the result of the first investigated state in the preceding section, and part of it in the upper portion and belongs to the second state, which is shown in Fig. 4a as dotted circles. Due to the specific shape of this structure, the approach of the metal contact to the absorbing layer reduces the parasitic losses and causes the light to return and trap in the active layer through the SPR enhancement of the metallic back-contact. As presented in the electric field profile, as expected considering the dimensions of the MNS ($>100$ nm), there is no confinement of light in the vicinity of Au surface. Consequently, the occurred SPR has no near-field effects, and it mainly has far-field effects and acts as a scatterer. Since the incident light is scattered in the longest wavelength of the studied spectrum, in shorter wavelengths, to the extent that light impacts the back-contact of Au, it scatters as well. Hence, one can say that the MNS acts only as a back scatterer and reflects most of the light (except for the part that is absorbed in the metal surface) to the first medium, at an angle equal to the incident angle according to Fresnel’s law. Overall, one can conclude that such a structure improved light entry, reduced parasitic losses, and trapped light in the cell.

**Figure 5:**
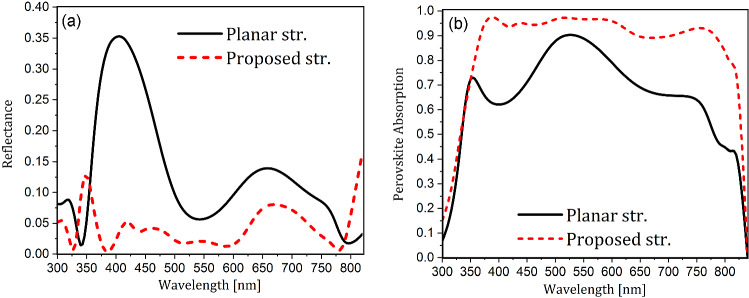
(**a**) The normalized reflectance and (**b**) absorption spectra of the planar and proposed structures.

Figure 5a,b illustrate the reflectance of the PSC and the absorption of its active layer for the planar and proposed structures. It is seen that the light reflectance has decreased almost in the whole spectrum in the proposed structure. As mentioned, the use of PDMS instead of glass on the cell surface reduces the reflection of the incident light due to its suitable refractive index. This is because, as mentioned earlier, its refractive index is lower than that of its underlying layer (ITO) and is closer to the external environment (air) with a refractive index of one. Therefore light better penetrates the structure. On the other hand, the non-planar structure causes the non-vertical movement of photons inside the PSC. Therefore, the light that enters the cell deviates from the cell’s perpendicular direction, and if it reflects internally at the interface of two layers and returns to the cell surface, it reaches it at an angle closer to the total reflection angle. Therefore, it is less likely to leave the cell, which leads to decreased reflection. However, in two parts of the wavelength range, there is a slight increase in the reflectance due to the change in the configuration of the cell, but it is small compared to the whole wavelength range and does not have much effect.

Due to the reduction of reflectance (Fig. 5a), and the trapping of light inside the cell, especially within the absorbing layer, according to the profile of the electric field (Fig. 4c), there is a significant increase in the perovskite absorption (Fig. 5b). Reductions in optical losses, including a decrease in parasitic losses in the red part of the spectrum ($\lambda$ $> 550$ nm) and a decrease in reflection losses in the blue part ($\lambda$ $< 550$ nm), causes 90% of the incoming light to be absorbed in the range of 400 to 750 nm. Therefore, one can see an increase in absorption throughout the proposed structure spectrum.

### Investigating the effects of reducing HTL thickness on cell function

The volume of materials in each layer in the proposed structure is equal to that in the planar structure, and only the thickness of the HTL in this structure is smaller. Therefore, to accurately compare these two structures in equal conditions of used materials, or in other words, to study the effect of reducing the thickness of HTL on the performance of the proposed cell, the output parameters of this cell with the ordinary volume of HTL (400 nm) must also be studied. However, as discussed in the structural analysis section, in practice, the deposition of a layer with this thickness on a textured substrate with such dimensions, destroys its angular formation and flattens the cell surface. Therefore, instead of examining the proposed structure with an ordinary thickness of HTL (400 nm), and comparing it with the main one (50 nm thick HTL), we study the planar structure with a thin HTL (the thickness that used in the proposed structure, which is 50nm) and compare it with the proposed structure. In such a case, the properties of the two structures are the same in terms of the materials used, and the comparison is reasonable.

**Figure 6:**
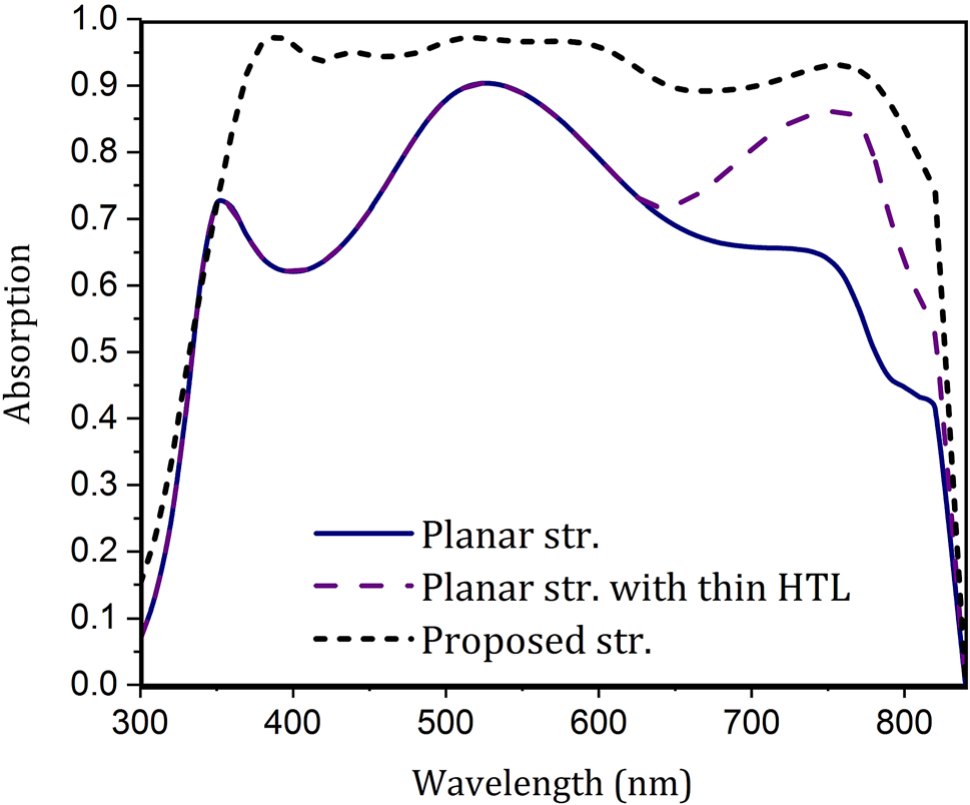
The normalized absorption spectrum for a conventional planar structure, the planar structure with a thin HTL (50 nm), and the proposed structure.

As shown in Fig. 6, reducing the thickness of the HTL in the planar structure increases the absorption only in the red part of the spectrum. Because, at shorter wavelengths, light is absorbed before it reaches the HTL. Therefore, the changes in the thickness of this layer do not affect this part of the spectrum. However, at higher wavelengths (the red light), the low energy of photons prevents them from being fully absorbed in the top layers. They thus enter the lower layer of CuSCN. In a conventional planar structure, the light that enters HTL is parasitically lost in this layer before reaching the back-contact of Au. But in the planar structure with thin HTL, reducing the thickness of this layer reduces light loss in this layer to a large extent. Therefore, at these wavelengths, the incoming light collides with the metallic back-contact and is scattered due to the SPR effect. In this planar structure, similar to the proposed structure, due to the large dimensions of the structure (assuming the smooth surface as a fragment of a circle with an infinite radius), the SPR has no near-field effects and only has far-field scattering effects, which causes light to return to the absorbing layer. As a result, decreasing the HTL thickness increases the absorption in the planar structure. However, the light absorption in this structure is still much lower than that of the proposed structure. Therefore, one can conclude that the high absorption of the proposed structure, while affected by the reduction of the thickness of this layer, is mainly due to the light trapping mechanism and the use of PDMS on the cell surface.

### Fabrication considerations of the proposed nanostructure of a PSC

**Figure 7:**
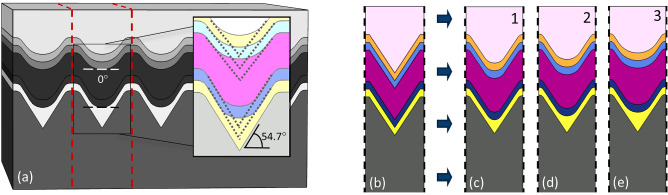
(**a**) The structure of the proposed PSC considering the conditions of fabrication processes. (**b**) The unit cell of previously studied structure, and (**c**–**e**) the configurations of the PSC unit cell concerning the fabrication conditions at three different curvature.

In the fabrication process, materials are not deposited with a perfectly smooth surface and uniform thickness throughout the cell, and depending on the type of fabrication process and the deposition conditions, they form differently. Since, on the one hand, the proposed structure has to be fabricated from the bottom up, and each layer should follow the shape of the previous layer, and on the other hand, the presented nanostructured PSC has angled configuration with a low width unit cell, after the fabrication process, it would not accurately form as proposed and would have a shape similar to the one shown in Fig. 7a, depending on the manufacturing conditions^47,49,50,53^.

According to the studies, by etching the crystal silicon (100), due to the high resistance of $<111>$ plane to etching, the textured silicon would be very similar to the presented structure with curvature angles of 54.7 degrees^34^. But by depositing the other materials, especially Au, as the first layer, the triangular parts would not copy, and the deposited layers would have a smooth curvature at their bottom, as shown in Fig. 7a with zero angles^34,47,49,50,53^. This increases with the deposition of each material, and each layer follows the shape of the previous layer to a smaller extent. As mentioned, that is the reason that in this structure, HTL not inserted with the thickness used in the planar PSC. In this case, after the deposition of such a thick layer, the structure would be almost flat. Figure 7c,d, and e show three different assumptions about the fabricated cell. In each case, the curvature of the angular section is reduced, and the form of textured silicon is less followed. The first fabricated conditions considered structure (FCCS) is very similar to that of the proposed one, and there is only a slight difference in the triangular part. This difference is slightly increased in the second FCCS and more in the third one. By studying these assumptions about the FCCSs (Fig. 7c–e) and comparing them with the proposed structure (Fig. 7b), the effects of fabrication conditions on cell performance can be examined.

**Figure 8:**
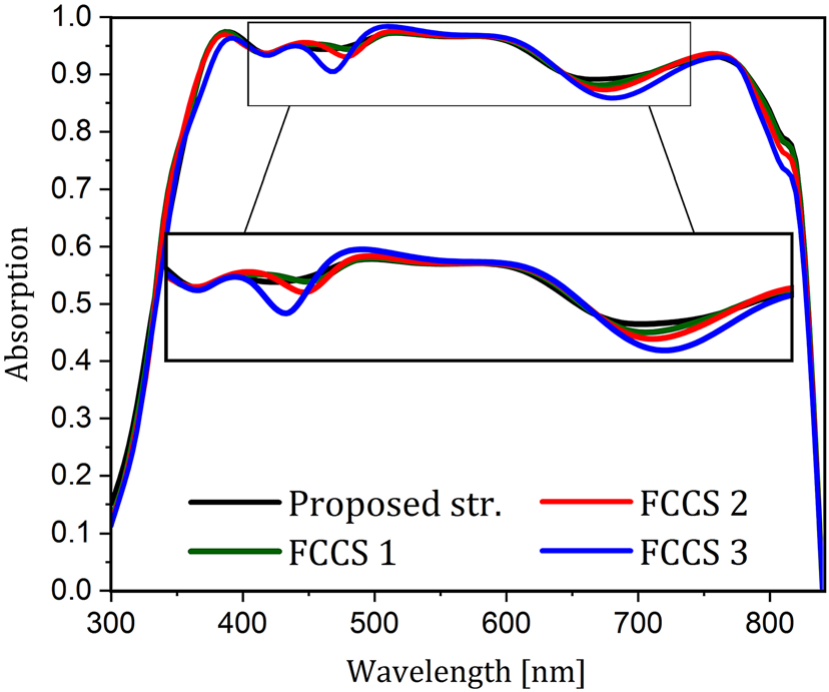
The normalized absorption spectra for the proposed structure and the three FCCSs.

Figure 8 shows the absorption spectrum of the active medium for the proposed nanostructure and three FCCSs shown in Fig. 7c,d, and e. According to this spectrum, the absorption of these structures is not very different from the proposed one, and there are only some changes in the range of 360 to 470, and 640 to 730 nm. In the first range, because of the higher energy of the photons, they are absorbed in the upper part of PSC and have no interaction with the lower layers of the perovskite. Therefore, the changes are due to the deformation of the ITO layer and the slight changes in the light path in each structure. In the second range of changes, the energy of the photons is low, so they penetrate more into the cell before being absorbed. As a result, the changes are due to the deformation of the metal contact, and the decrease in light trapping. Since in each structure, the curvature of the triangular part is reduced, the probability of light escaping from the cell, and being scattered by SPR towards the cell surface is increased as well. There is, thus, a slight decrease in the absorption of each FCCS in this range. Overall, the absorptions of these structures are not very different, and they all absorb almost the same amount of light.

### Electrical performance of the proposed nanostructure and three FCCSs of PSCs

**Figure 9:**
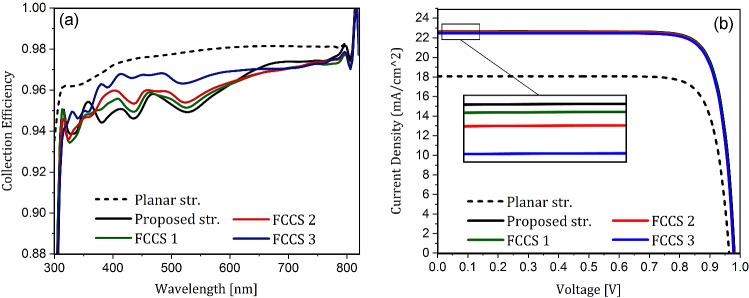
(**a**) The normalized collection efficiency spectra, and (**b**) the current-voltage characteristics for the planar PSC, the proposed structure, and the three FCCSs.

In this work, the collection efficiency spectrum is used to investigate the rate of transferred electrons and holes to the charge transport layers. This parameter shows the ability of carrier extraction and its transportation in a solar cell and is obtained by dividing the number of collected carriers by the contacts to the number of photo-generated electron-hole pairs at each wavelength. Fig. 9a shows the normalized collection efficiency spectrum for the planar PSC, the proposed structure, and the three FCCSs. As expected, in all PSCs, by increasing the wavelength, the carrier collection rate is improved. This is because at longer wavelengths, due to the reduction of photons energy according to the relation of $E=\frac{hc}{\lambda }$, the photons travel a longer path before being absorbed. As a result, the electric field inside the absorbing layer becomes distributed, and the extraction of generated carriers becomes easy due to their proximity to the interfaces of both carrier transport layers. According to the figure, planar PSC is more efficient in collecting the carrier than the proposed structure and its FCCSs. It can be attributed to the distribution of the electric field inside the solar cell. As seen in Fig. 4b,c, unlike the planar structure, in the proposed architecture, the distribution of the electric field in the active layer is not uniform. Consequently, the photo-generated carriers are in the specific sites of the active layer, and due to the angular configuration of the proposed structure, they must travel a longer path to reach the charge extraction layers. In other words, because of the light trapping mechanism of this PSC, the effective interface of the absorbing layer with the carrier transport layers at a constant unit cell width is reduced, making carrier collection difficult. On the other hand, collection efficiency improves to some extent by reducing the curvature of the triangular part in FCCSs. According to Fig. 9a, among the proposed structure and its fabrication considered PSCs, the third FCCS has the best collecting characteristic. Again, this result can be attributed to the effective interface of layers and the carrier transport path. By reducing the curvature of the angular part of the cell, due to the proximity of their generated sites to the interfaces of the transport layers, the extraction of the produced carriers is facilitated. The fluctuating trends of the collection efficiency spectrum in the proposed structure and its FCCSs are also due to differences in the distribution of the electric field at each wavelength.

Figure 9b shows the characteristics of the current density in terms of voltage for a planar PSC, the proposed structure, and three FCCSs of a proposed PSC. As expected, the reduction in reflection and parasitic losses, and increased light absorption of the perovskite layer due to light trapping results in an increase in the J_sc_ in the presented structure compared to the planar PSC. But since the constituents of the PSC and the thickness of the active layer remains unchanged, filling factor does not change significantly. However, there is a slight change in V_oc_. This is partly due to the increase in current density. Because it has caused in higher voltage, the cell current (J_dark_) is equal to the carrier generation current (J_sc_). That is the reason that the third FCCS, although having a better collection efficiency, has slightly smaller V_oc_ than that of the main proposed structure and the other FCCSs . On the other hand, due to the reduction in the thickness of the HTL, the recombination in this material is decreased, leading to a higher V_oc_. Considering the limited diffusion length of the holes, the reduction of the current path reduces the carrier losses during transportation in this layer. Overall, these two factors improve the V_oc_ in this structure.

**Table 1:** The used electrical parameters in the PSCs simulations.

Parameter	$\mathbf{TiO} _\mathbf{2 }$	Perovskite	CuSCN
$N_{C}$ (cm$^{-3}$)	$1\times 10^{19}$	$5.41\times 10^{19}$	$2.51\times 10^{19}$
$N_{V}$ (cm$^{-3}$)	$1\times 10^{19}$	$1.66\times 10^{19}$	$1.79\times 10^{19}$
$N_{A}$ (cm$^{-3}$)	0	$5\times 10^{13}$	$5\times 10^{18}$
$N_{D}$ (cm$^{-3}$)	$5\times 10^{18}$	0	0
$\varepsilon _{\mathbf{r}}$	9	6.5	10
*X* (eV)	4	3.93	1.9
$E_{g}$ (eV)	3.2	1.5	3.4
$\tau _{n}/\tau _{p}$ (ns)	5/2	8/8	5/5
$\mu _{n}/\mu _{p}$ (cm^2^/V s)	20/10	50/50	$1\times 10^{-4}/0.01$

**Table 2:** The short-circuit current density (J_sc_), the open-circuit voltage (V_oc_), the filling factor (FF), and the power conversion efficiency (PCE) for the planar, the presented, and three fabrication conditions considered structures of PSCs.

Structure	Planar Str.	Proposed Str.	FCCS 1	FCCS 2	FCCS 3
$\mathbf{J} _{\mathbf{sc}}$ (mA/cm^2^)	17.51	22.45	22.39	22.28	22.34
$\mathbf{V} _{\mathbf{oc}}$ (V)	0.96	0.98	0.98	0.98	0.97
**FF** (%)	82.42	83.13	83.09	83.06	82.92
**Efficiency** (%)	13.86	18.29	18.19	18.08	18.04

According to the J-V characteristics presented in Table 2, the J_sc_ of the FCCSs, such as their absorption, do not differ much from the main presented structure. This indicates that if the proposed structure is fabricated, it would exhibit high effieincy even though it may not be identical to the design proposed structure. According to the Table 2, the J_sc_ of the proposed structure is 22.45 mA/cm^2^, which is increased by 28% compared to the planar structure. The V_oc_ is also increased and reaches a value of 0.98 volts. The J_sc_ of the FCCSs are also almost equal to the main nanostructured PSC, and in the worst case, has a value of 22.28 mA/cm^2^ for the second FCCS. The conversion efficiency is also increased by 32%, from 13.86% for the planar structure to 18.29% for the presented structure.

## Discussion

In the present study, a coupled optical-electrical simulation has been performed to obtain the optical spectra and electrical parameters of the PSCs. First, the light loss factors in the planar architecture of a PSC have been analyzed, and a nanostructured solar cell on a textured silicon substrate has been presented to reduce these losses. The results have shown that the use of PDMS instead of glass in the proposed structure reduces the reflection loss of the cell. Furthermore, the perovskite absorption due to the light trapping in the active layer, and the far-field scattering enhancement of SPR have been found to increase dramatically. The effects of reducing the HTL thickness on the perovskite absorption have been investigated, and the mechanism of SPR on the surface of the metallic back-contact has been determined. The possible formation of the presented PSC according to the fabrication process has also been considered, and the optical absorption in these nanostructures has been compared with that of the main structure. The perovskite absorption in these structures have been found to be not very different from the main structure, and the PSCs have shown significant efficiencies. In the final part of the research, the current-voltage characteristics of the main proposed structure and three FCCSs have been investigated. The findings have revealed that due to the increased absorption almost in the entire spectrum, and the improvement of carriers’ transportation, J_sc_ and V_oc_ increase significantly. As a result, the power conversion efficiency of the PSC was seen to increase from 13.86% to 18.29%. Based on the calculations, the PCE in the most different case of the FCCS compared to the main structure has been shown to have a value of 18.04%, which has still a notable efficiency improvement.

## Methods

Since a solar cell is a photovoltaic device, its optical and electrical physics must be studied simultaneously. In the optical part of the simulation, the behavior of the cell exposed to sunlight and the resulting photo-generated carriers are investigated. In the electrical section, the characteristics of the PSC in the flow of these generated carriers are examined.

**Photo-generated carriers measurments.** In the optical simulations part, the Maxwell’s equation (Eqs. 1 and 2) is used to calculate the electric field of the propagated light inside the cell, which is emitted by the incoming light source.

1 $$\begin{aligned}&\frac{\partial H}{\partial t} = \frac{-1}{\mu } \nabla \times E \end{aligned}$$

2 $$\begin{aligned}&\epsilon \frac{\partial E}{\partial t} = - \nabla \times H - \sigma E \end{aligned}$$

In this equation, *H* is the magnetic field, $\mu$ is permeability, *E* is the electric field, $\varepsilon$ is permittivity, and $\sigma$ is the electric conductivity. For the simulated unit cell of the investigated solar cells, a periodic boundary condition is implemented to all sides of cell’s layers except the Au contact. Perfect electric conductor as boundary condition is used for this part in order to consider the reflected light from its surface. The light source is a plane wave and is placed outside the cell in a medium with air properties to calculate the reflection of the cell surface as well. The input light power is based on the standard AM 1.5 and is applied over the wavelength range of the perovskite absorption with a resolution of 10 nm.

Using Eqs. 1 and 2, the electric field is obtained in the whole domain at each wavelength. With the measured value of the electric field inside the perovskite, the amount of photo-generated electron-hole pairs in the absorbing medium in each wavelength is calculated as follows:

3 $$\begin{aligned} G_{\lambda }(\lambda )=\frac{\varepsilon ^{\prime \prime } |E(\lambda )|^2}{2h} \end{aligned}$$

where $\varepsilon ^{\prime \prime }$ is the imaginary component of relative permittivity, and *h* is the Plank’s constant. By the integral of $G_{\lambda }$ at the entire wavelength range, the total carriers generated by the simulated sunlight ($G_{{\mathrm{total}}}$) is calculated as follows:

4 $$\begin{aligned} G_{total}=\int _{\lambda _{min}}^{\lambda _{max}} G_{\lambda }(\lambda )\;\mathrm {d}\lambda \end{aligned}$$

**Absorption measurments.** Absorption of each layer and reflection from the PSC surface are obtained using the scattering parameters^58^:

5 $$\begin{aligned} Absorption= & {} 1-(|S_{11}|^2+|S_{21}|^2) \end{aligned}$$

6 $$\begin{aligned} Reflection= & {} |S_{11}|^2 \end{aligned}$$

In calculating these parameters, the power of the input light is unity. This is because the elements of the scattering matrix only show the absorption, transmission, and reflection percentages of the incoming light. Therefore, the input power does not affect the calculation. The complex refractive indices in terms of wavelength for the investigated solar cell constituents, namely TiO_2_, perovskite (CH_3_NH_3_PbI_3_), CuSCN, and Au are obtained from previous calculations^59–62^. However, for ITO, two different refractive indices are considered in the cases of ITO on glass or PDMS^63^.

**Electrical characterization.** Using $G_{{\mathrm{total}}}$, one can obtain the electrical properties of the photovoltaic cell exposed to light. The following equation is employed to calculate the current density - voltage characteristic of the PSCs:

7 $$\begin{aligned} J(V)=J_{sc}(G_{total})+J_{dark}=J_{sc}(G_{total})-\left| J_{0}\left( \left( exp\frac{eV}{nKT}\right) -1\right) \right| \end{aligned}$$

where *e* is the charge of an electron, *n* is an ideality factor that depends on the type of the matter, *K* is the Boltzmann’s constant, and *T* is the temperature. According to this equation, the current of a cell consists of two components of $J_{{\mathrm{sc}}}$ and $J_{{\mathrm{dark}}}$ that flow in opposite directions in the cell. $J_{{\mathrm{sc}}}$ is caused by light propagation in the cell, and consequently, generated carriers by the absorbed photons. But $J_{{\mathrm{dark}}}$ does not relate to the carriers generated by incident light and is a function of the voltage applied to the two ends of the cell. Both of these currents are obtained using Poisson and continuity equations. The calculations of the electrical part of the cell are made only for three layers of TiO_2_ and CuSCN as the electron and hole transport layers, respectively, and perovskite as the absorbing layer. Ideal ohmic for the upper contact of ITO and Schottky condition with the surface recombination speed of 10^7^ cm/s for the lower contact of Au are considered. The electrical parameters used are presented in Table 1^11,38,64–73^. Here, $N_{\mathrm{C}}$ and $N_{\mathrm{V}}$ are effective density of states of conduction and valence bands, $N_{\mathrm{A}}$ and $N_{\mathrm{D}}$ are acceptor and donor densities, *X* is electron affinity, $E_{\mathrm{g}}$ is bandgap energy, $\tau _{n}$ and $\tau _{p}$ are electron and hole lifetimes, and $\mu _{n}$ and $\mu _{p}$ are electron and hole mobilities, respectively. The computational method used in this work is the FEM. It is a mathematical analysis technique that is widely used to solve a variety of engineering problems.

## Data Availability

The data generated and analyzed during the study are available from the authors on reasonable request.
